# Patient Perception of the Relevance of Quality-of-Life Questionnaires in Waldenström Macroglobulinaemia: An International Survey

**DOI:** 10.3390/cancers17223609

**Published:** 2025-11-08

**Authors:** Jahanzaib Khwaja, Sotirios Bristogiannis, Ibrahim Tohidi-Esfahani, Yadanar Lwin, Nicole Japzon, David Young, Sossana Delibasi, Shirley D’Sa, Judith Trotman, Charalampia Kyriakou

**Affiliations:** 1 Department of Haematology, University College London Hospital, London NW1 2BU, UK; j.khwaja@nhs.net (J.K.);; 2 Department of Haematology, Evangelismos Hospital, 10676 Athens, Greece; 3 Department of Haematology, Concord Repatriation General Hospital, University of Sydney, Concord 2139, Australia; 4 Department of Haematology, Nottingham University Hospital NHS Trust, Nottingham NG7 2GT, UK; 5 WMozzies, Australian Affiliate International Waldenstroms Macroglobulinaemia Foundation, Sydney, Australia

**Keywords:** quality of life, Waldenstrom macroglobulinaemia, lymphoplasmacytic lymphoma

## Abstract

Waldenström Macroglobulinaemia is an indolent disease which is recognised as contributing to a diverse range of disabling symptoms which can impact quality of life. Little data are available in evaluating patient and healthcare professional views on quality-of-life questionnaires (QLQ) used to evaluate quality of life in Waldenström Macroglobulinaemia. We aimed to assess patient perception of their disease and the relevance of the currently employed QLQs in practice by patients and healthcare professionals. One-hundred-and-twenty patients (22% treatment-naïve) and thirty-four healthcare professionals completed an international survey to understand their perception of the relevance of the currently employed QLQ. Out of 415 questions, 257 questions were classified by patients as relevant. Five themes were discerned as relevant: fatigue, mobility, dyspnoea, paraesthesia, pain in the hands and feet, sleep (physical health limitations); contentment (psychological impact); social and family support. The following were considered irrelevant: lumps or swelling, dry mouth, trouble hearing, sexual dysfunction (physical symptoms); post-traumatic stress (psychological impact); education and training and financial support. Healthcare professionals identified EORTC QLQ-C30 as the most commonly used instruments for Waldenström Macroglobulinaemia trials.

## Simple Summary

We conducted an international survey of 120 patients and 34 healthcare professionals to explore how well existing quality-of-life questionnaires capture what matters most to people with Waldenstrom macroglobulinaemia. Patients highlighted fatigue, mobility, pain, tingling, breathlessness, sleep issues, emotional wellbeing, and family support as the most relevant topics, while many questions about other issues such as post-traumatic stress, education and training and financial support were seen as less relevant. Commonly used questionnaires do not fully reflect patients’ real experiences, suggesting the need for WM-specific tools.

## 1. Introduction

Waldenström Macroglobulinaemia (WM) is a low-grade lymphoproliferative disorder characterised by an IgM monoclonal protein and lymphoplasmacytic bone marrow infiltrate [1]. It has a recognised diverse range of potentially disabling associated conditions including peripheral neuropathy, cryoglobulinemia and cold agglutinins which can impact quality of life (QoL). Median survival is over a decade [2] and despite expanding available therapies, complete haematological responses remain rarely achieved. As a chronic condition, there has been focus on QoL. Beyond standardised haematological responses, regulatory bodies and patient advocacy groups highlight the need to understand patient-reported symptoms as a priority [3].

A number of QoL questionnaire instruments (QLQ), are utilised to understand the impact of the disease. These non-disease-specific QLQs are derived from generic cancer cohorts in a historic treatment era [4], so they may not be relevant to issues faced by patients undergoing modern treatments. WM may be associated with unique complications which may not be covered by generic cancer cohorts. A meta-analysis of 10 studies of QLQs in WM showed limited reliability, validity and responsiveness assessment for WM in accordance with gold standards [5]. It should be noted that healthcare professional (HCP) observation of symptom severity and perception of disease may be disparate from self-reported patient assessment [6]. We therefore aimed to assess patient perception of their disease and the relevance of the currently employed WM QLQs in practice by patients and HCPs.

## 2. Methods

Patients ≥ 18 years with WM were invited via email to complete an anonymised English-language cross-sectional online survey to rate the relevance of QLQ used in WM, distributed via SurveyMonkey. Patient support groups across Europe, Australia and the United States were contacted to support the dissemination of the survey, coordinated by the University College London Hospital (United Kingdom), the data controller for the UK Rory Morrison Registry, alongside WMozzies Registry (Australia), the European Consortium for Waldenstrom Macroglobulinaemia and the International Waldenstrom’s Macroglobulinaemia Foundation. Clinicians within these networks were additionally invited to complete the healthcare professionals’ views. Patient representatives from WM support groups were involved in the development of the survey to ensure that structure, style and language were suitable, whereas the piloting of this survey and contact details were provided for support. Patients with cognitive impairments which hampered the completion of the survey were excluded. The 14 most commonly used QLQs identified from our systematic literature review on patient-reported outcomes were used based on the previous literature [5]. The survey was conducted in accordance with the Declaration of Helsinki.

Patient self-reported demographics, current and previous symptom burden and treatment preferences were collected. Patients were then asked to grade perception of individual QLQ questions on a 5-point Likert scale: 0 ‘not relevant’, 1 ‘slightly relevant’, 2 ‘relevant’, 3 ‘fairly relevant’, and 4 ‘very relevant’.

Four-hundred-and-fifteen questions from the following QLQs were included: EORTC QLQ-C30; EQ-5D-5L; FACT-G; FACIT-F; FACT-An; FACT-GOG/NTx; FACT-Lym; SF-36; HADS; DASS-21; QLACS; IES; FSS; IPA (Table 1). Items for which >50% of patients selected options 1–4 were considered relevant. Duplicate questions and similar questions considered redundant were excluded (such as ‘have you had pain?’ ‘I have aches and pains’ ‘I have pain and discomfort’). For descriptive purposes, questions were categorised into broad themes by three investigators: physical health limitations (30 issues), psychosocial impact (29 issues), family impact (2 issue), social impact (5 issues), educational impact (1 issue) and financial impact (2 issues).

Patients and a convenience sample of healthcare professionals (including physicians, nurses, psychologists and support workers) were (separately) invited to express in free text any other QoL issues that they felt should be addressed but were not covered in the survey. Descriptive data were analysed with number, percentage and medians. Paired proportions were compared using McNemar’s test and reported using STATA v18 (STATAcorp, College Station, TX, USA). The free text was summarised in a word cloud.

## 3. Results

A total of 120 patients responded, 87 from the United Kingdom, 20 from Australia and one each from France, Germany, and the USA (Table 2). The median time to complete the survey was 15 min. In total, 53% were male and 47% were female. A total of 4% of responders were <50 years, 18% were 51–60 years, 41% were 61–70 years, and 37% were >70 years. Meanwhile, 110 patients were of white ethnicity, 2 were Hispanic, 1 each was Asian and other, 6 gave no response. In addition, 62% were married, 18% single, 20% other, 68% were retired and 23% were currently employed. ECOG performance status was 0 (50%), 1 (43%), 2 (4%) or 3 (3%).

WM was diagnosed greater than 5 years prior to the time of survey completion in 52%, 1–5 years in 37% and <1 year in 12%. Of the total cohort, 22% of respondents were treatment-naïve whilst for the treated patients, the median number of therapy lines was 1 (range 1–7). A total of 18% had 2 and 17% had ≥3 lines of therapy. In total, 93 patients were treated with the following prior therapies: 31 with bendamustine-rituximab (BR), 23 with dexamethasone-rituximab-cyclophosphamide (DRC), 14 with rituximab, 9 with ibrutinib, 2 with zanubruinib, 1 with acalabrutinib, 7 with RCHOP (rituximab-cyclophosphamide-doxorubicin-vincristine), 5 with fludarabine-based therapy, 1 with R-cladribine, 3 with bortezomib-based therapy, 1 with nivolumab, 1 with pembrolizumab, 2 with chlorambucil, 4 with ifosfamide-based, 6 with autologous stem cell transplant and 11 underwent clinical trials. Current therapy at time of completion included the following (*n* = 43): BTKi (Bruton tyrosine kinase inhibitors: 16 zanubrutinib, 12 ibrutinib, 1 ibrutinib-rituximab, 1 acalabrutinib), 8 BR, 4 DRC, and 1 rituximab.

**Figure 1:**
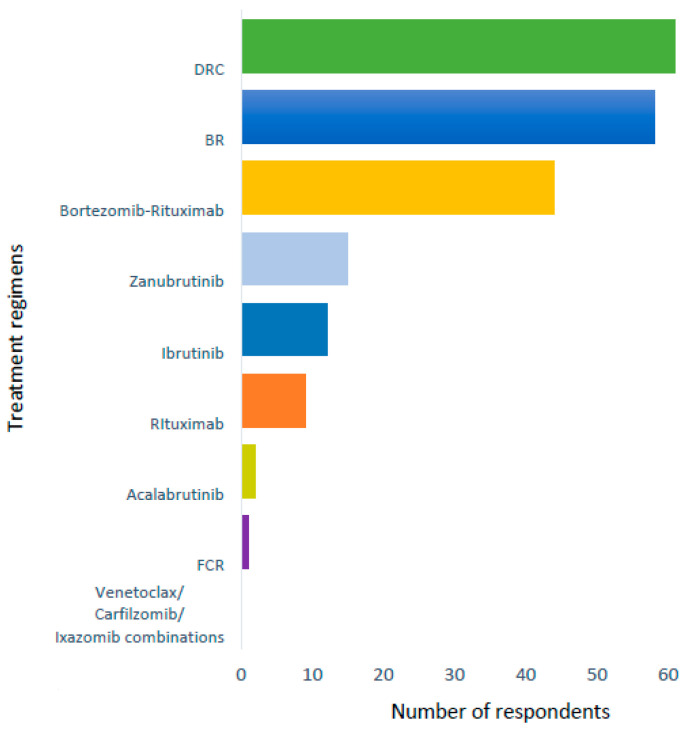
Patient preference of treatment regimens.

**Figure 2:**
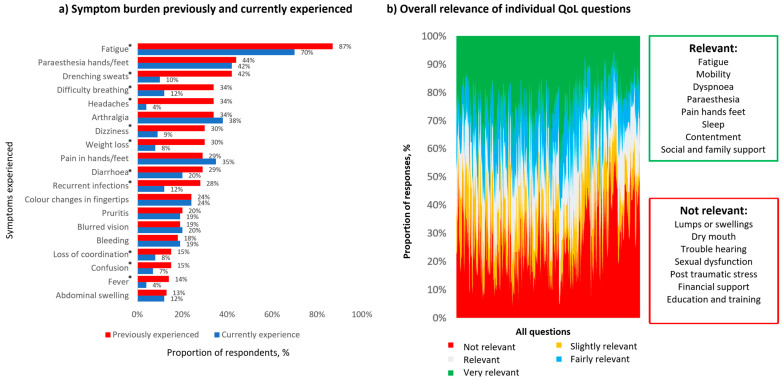
(**a**) Symptom burden previously and currently experienced. (**b**) Overall relevance of individual QoL questions. * <0.05 by McNemar’s test for paired proportions to assess differences in current and previous symptoms.

Patients were asked to rank their preference of treatments they had received. DRC and BR were considered the most tolerable regimens for patients when asked to rank their preference out of possible regimens (Figure 1). The most prevalent symptoms patients had previously experienced were fatigue (87%), paraesthesia (44%) and sweats (42%). Fatigue remained the most prominent current symptom (71%) with a similar proportion of paraesthesia (42%) but smaller proportion of sweats (10% vs. 42%), weight loss (30% vs. 8%) and fever (14% vs. 4%) when compared with previously experienced symptoms (Figure 2).

Four-hundred-and-fifteen questions were graded. One-hundred-and-twenty-nine questions were duplicates across different QLQs (127/129 across FACT-G, FACIT-F, FACT-An, and FACT/GOG-NTx, FACT-Lym) leaving 286 questions. A total of 257 questions were relevant (>50% total slightly relevant/relevant/fairly relevant/very relevant), spread across questionnaires, and the remaining 29 questions were considered irrelevant (Figure 2b).

The most significant items discerned as relevant (>50% with response 1–4) within all themes were as follows: fatigue, mobility, dyspnoea, paraesthesia, pain in the hands and feet, sleep (physical health limitations); contentment (psychological impact); social and family support. The following were considered irrelevant: lumps or swelling, dry mouth, trouble hearing, sexual dysfunction (physical symptoms); post-traumatic stress (psychological impact); education and training and financial support.

Thirty-four healthcare professionals (HCPs) completed the survey (26 physicians, 3 nurses, 1 pharmacist, and 3 other allied healthcare professional), from the United Kingdom (*n* = 14), Australia (*n* = 2), Greece (*n* = 3), Italy (*n* = 5), Netherlands (*n* = 2), Austria (*n* = 2), Germany (*n* = 2), France (*n* = 2) and Portugal (*n* = 1), with years of experience as follows: 0–5 years (*n* = 11), 5–10 years (*n* = 6), and >10 years (*n* = 16). HCPs were asked which instruments they use for WM trials. The majority utilised EORTC QLQ-C30 (74%, 23/31) or EQ-3D-3L (48%) and <30% all other QLQs.

**Figure 3:**
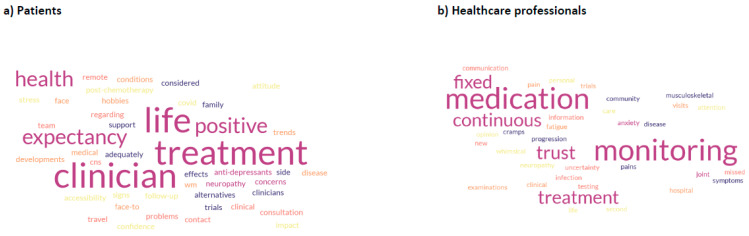
View of issues not explored in QLQs, mentioned by participants (**a**) Patient views (**b**) Healthcare professional views.

An additional free text view of patients and HCPs was elicited. Thirty-two patient responses and 30 HCP responses are summarised in a word cloud (Figure 3). The most frequent patient responses centred on treatment, interaction with clinicians and leading a positive life. This compared with medication monitoring, fixed vs. continuous therapy and trust for HCPs.

## 4. Discussion

Patient-centred outcomes are frequently cited by advocacy groups and regulatory authorities [7]; however, scant data are available evaluating patient views on QLQ in the current treatment era in WM. There have been no data assessing patient views on existing QLQs. QLQs are designed as standardised, validated tools to measure health and wellbeing and may be used to monitor conditions longitudinally. Recently disease-specific QLQs have been developed for other chronic haematological disorders including chronic myeloid leukaemia [8].

We demonstrated that relevant issues are fragmented across questionnaires and there is a redundancy in QLQs when aggregated together. In our cohort, fatigue was the dominant feature, considered the most relevant theme in QLQs and experienced most frequently during the disease course, including in the treatment-naïve setting. Exploratory end points in clinical trials have frequently reported this as a key feature, and haematological responses correlated with improvements in the head-to-head ASPEN study comparing ibrutinib to zanubrutinib [9]. Fatigue is multifaceted and may manifest in relevant symptoms including shortness of breath, reduced physical function, mobility and ability to maintain social and family roles. In addition, the relevant items of dyspnoea and headache in WM may be associated with hyperviscosity.

Neuropathic symptoms were also featured as relevant. This may manifest in a number of IgM-associated conditions. IgM entities contributing to neuropathy include anti-MAG neuropathy, AL amyloidosis, and cryoglobulinemia as well as, infrequently, WM infiltration into the central nervous system (Bing–Neel syndrome). Neuropathic symptoms featured in similar proportions of previously experienced and currently experienced symptoms, which presumably reflects the chronic nature of this disability. A discrete choice experiment including 214 patient responses from the Netherlands demonstrated that avoiding neuropathy was the most important adverse event consideration when selecting treatment [10], more so than extreme fatigue, nausea and vomiting. Participants were willing to trade 6.5% in efficacy to avoid neuropathy. Traditionally treatment-related neuropathy had been attributed to bortezomib, particularly when administration was intravenous and twice weekly, although it may be considered more tolerable now [11].

Patients highlighted a preference for traditional fixed-duration chemoimmunotherapy (BR, DRC and bortezomib-based) above BTKis. The consideration of fixed-duration vs. continuous therapy was the greatest healthcare professional concern in the free text. Future trials investigating fixed-duration novel combinations achieving deep remissions have been recommended in consensus guidelines [12] and would be welcomed.

WM typically affects older adults, and age is robustly prognostic for overall survival, forming a key part of the WM staging system [13]. Social and family support featured as important relevant functional scales. Conversely, our cohort considered financial support, training, education, sexual dysfunction and post-traumatic stress as non-relevant issues, which we speculate may be more pertinent to a selective proportion of younger patients. Younger patients with WM have excellent outcomes and may benefit from age-adjusted scores [14]. This highlights the potential limited value of the IES (Impact of Event Scale) and QLACS QLQs measuring these domains.

Healthcare professionals identified EORTC QLQ-C30 as the most commonly used instruments for WM trials. United Kingdom registry data from 155 patients across 58 questions (including EORTC QLQ-C30) identified that over two-thirds of items failed to elicit a notable median response [15]. Often, large proportions of health limitations and symptoms are not directly assessed in individual QLQs [5]. With a changing treatment landscape [16], such tools should be sequentially assessed over time. The only literature review reporting on QLQs in WM included 10 studies and showed EORTC QLQ-C30 alone to have acceptable content validity, whereas other selective QLQs delved further into specific aspects only—such as emotional burden. In this survey EORTC WLW-C30 included both relevant (fatigue, dyspnoea) and irrelevant (hearing, dry mouth, financial) issues suggesting opportunity for refinement. ED-5D-5L measured dimensions considered relevant, including mobility, self-care and usual activities and FACIT-F, FACT-GOG/NTx captured fatigue and neuropathy, which align with relevant patient issues.

## 5. Limitations

We acknowledge clear limitations of such a survey including selection bias which is inherent in voluntary surveys. The median time of completion was 15 min, suggesting that this was not burdensome. The short time frame may, however, reflect limited patient engagement, but we are mindful that, in order to capture the most representative group of patients, prolonged time may have been inhibitory for participation given the extensive QLQs involved. The overwhelming majority was white, which is disparate from the reported ethnicity in WM. Patient selection is critical to ensure a representative WM questionnaire is created in the future, and although patient advocate groups were involved in the design and dissemination to ensure a broad group were targeted across different countries, we acknowledge this as a key challenge in a rare disease. The abilities to know that patients fully comprehend questions, recall the requested information and reliably report their responses were not formally assessed as this was a voluntary self-directed survey. Because of limited numbers, we were unable to stratify differences in responses by baseline characteristics including age or treatment. Too few HCPs responded to the survey to make strong conclusions.

## 6. Conclusions

Our current work reinforced the data of most prevalence symptoms including fatigue and paraesthesia, social and family roles. Relevant patient issues may be fragmented across QLQs and, therefore, a WM-specific QLQ would be welcomed to explore different subgroups and to take into account QoL issues acknowledged by patients.

## Figures and Tables

**Table 1:** Quality-of-life instruments.

Quality-of-Life Instruments	Abbreviation
EORTC Core Quality-of-Life-questionnaire	EORTC QLQ-C30
EuroQol 5-Dimension 5-level	EQ-5D-5L
Functional Assessment of Cancer Therapy—General	FACT-G
Functional Assessment of Chronic Illness Therapy—Fatigue	FACIT-F
Functional Assessment of Cancer Therapy—Anaemia	FACT-An
Functional Assessment of Cancer Therapy/Gynaecologic Oncology Group—Neurotoxicity	FACT-GOG/NTx
Functional Assessment of Chronic Illness Therapy—Lymphoma	FACT-Lym
36-Item Short Form Health Survey questionnaire	SF-36
Hospital Anxiety and Depression Scale	HADS
Depression, Anxiety and Stress Scale—21 Items	DASS-21
Quality of Life in Adult Cancer Survivors	QLACS
Impact of Events Scale	IES
Fatigue Severity Scale	FSS
Interpretative Phenomenological Analysis	IPA

**Table 2:** Demographics and treatment history of surveyed patients with WM.

Characteristic, *n* (%)	*n* = 120 ^Ƚ^
Age, years (*n* = 114) <50 51–60 61–70 >70	5 (4) 20 (18) 47 (41) 42 (37)
Gender (*n* = 114) Male Female	60 (53) 54 (47)
Country (*n* = 110) UK Australia France Germany USA	87 (79) 20 (18) 1 (1) 1 (1) 1 (1)
ECOG Performance status (*n* = 113) 0 1 2 3	56 (50) 49 (43) 5 (4) 3 (3)
Prior therapy exposure (*n* = 93) BR DRC Rituximab single-agent BTKi * RCHOP Fludarabine-based Clinical trial Mel-ASCT Bortezomib-based Other	31 (33) 23 (25) 14 (15) 12 (13) 7 (8) 5 (5) 11 (12) 6 (6) 3 (3) 9 (10)
Current therapy (*n* = 43) BTKi BR DRC Rituximab	30 ^ǂ^ (70) 8 (19) 4 (9) 1 (2)

BR, bendamustine-rituximab; DRC, dexamethasone-rituximab-cyclophosphamide; BTKi, Bruton tyrosine kinase inhibitors; RCHOP, rituximab-cyclophosphamide-doxorubicin-vincristine; Mel-ASCT, melphalan conditioned autologous stem cell transplant. * ibrutinib (*n* = 9), zanubruinib (*n* = 2), acalabrutinib (*n* = 1); ^ǂ^ zanubrutinib (*n* = 16), ibrutinib (*n* = 12), ibrutinib-rituximab (*n* = 1), acalabrutinib (*n* = 1). ^Ƚ^ refers to the total number of responders. The number of responders per question is outlined in the characteristics due to missing responses.

## Data Availability

The datasets analysed during the current study are available from the corresponding author on reasonable request.
